# Supplementary material for: Targeting BRAF Activation as Acquired Resistance Mechanism to EGFR Tyrosine Kinase Inhibitors in EGFR-Mutant Non-Small-Cell Lung Cancer
Source: Pharmaceutics. 2021 Sep 15;13(9):1478. doi: 10.3390/pharmaceutics13091478 (PMC8471192; doi:10.3390/pharmaceutics13091478)
Supplement: Supplementary file 1 [file pharmaceutics-13-01478-s001.zip › pharmaceutics-1340846 - supplementary.pdf]

pharmaceutics

MDPI

Supplementary Materials: Targeting BRAF Activation as Acquired Resistance Mechanism to EGFR Tyrosine Kinase Inhibitors in EGFR-Mutant Non-Small-Cell Lung Cancer

Frank Aboubakar Nana [1,2] and Sebahat Ocak

Table S1. Main clinical trials performed with third-generation EGFR tyrosine kinase inhibitors and BRAF +/- MEK inhibitors in advanced-stage NSCLC.

Compound	Company	Clinica trial																	Drugs No Longer in Active Clinical Development
caption	caption	Phase	Clinical Trials.gov identifier	Name	Indication	N	Drugs	ORR, % (95% CI)	OR (95% CI)	*P* value	Median PFS, Months (95% CI)	HR (95% CI)	*P* value	Median OS, Months (95% CI)	HR (95% CI)	P value	Recruitement Status	References	caption
**Third-generation EGFR TKIs**																			
Abivertinib or avitinib (AC0010)	Hangzhou ACEA Pharmaceutical Research Co.	1	NCT02330367		*EGFR*+ NSCLC, after previous EGFR TKI	52	Abivertinib 50, 100, 200, 350, 500, 550, 600 mg BID (RP2D: 300 mg BID)	36.5									Unknown	[1]	
		2	NCT03300115		*EGFR* T790M+ NSCLC, after previous EGFR TKI	222	Abivertinib 300 mg BID										Unknown		
		3	NCT03058094	AEGIS-1	*EGFR* T790M+ NSCLC, after previous EGFR TKI	0	Arm 1: Abivertinib 300 mg BID										Withdrawn		
							Arm 2: Cisplatin-pemetrexed												
Alflutinib (AST2818)	Shanghai Allist Pharmaceuticals	1	NCT02973763		*EGFR*+ NSCLC, after	14	Alflutinib 20, 40, 80,	50.0									Active, not recruiting	[2]	

					previous EGFR TKI		160, 240 mg QD								
		1/2	NCT03127449		*EGFR*+ NSCLC, after previous EGFR TKI	116	Alflutinib 80, 160 mg QD (RP2D: 80 mg QD)	76.7 (68.0–84.1)				11.1 (9.6-NR)	Active, not recruiting	[2]	
		2	NCT03452592	ALSC003	*EGFR* T790M+ NSCLC, after previous EGFR TKI	220	Alflutinib 80 mg QD	74.1 (67.8–79.7)				9.6 (8.2–9.7)	Unknown	[3]	
Almonertinib (HS-10296)	Jiangsu Hansoh Pharmaceutical Co.	1	NCT02981108		*EGFR*+ (including T790M+) NSCLC, after previous EGFR TKI	120	Almonertinib 55, 110, 220, 260 mg QD (RP2D: 110 mg QD)	50 (41–59) overall, 52 (42–63) in 94 patients with T790M+	9.6 (8.3–11.1), 11.0 (9.5-NR) in 94 patients with T790M+				Unknown	[4]	
		2	NCT02981108	APOLLO	*EGFR*+ (including T790M+) NSCLC, after previous EGFR TKI	244	Almonertinib 110 mg QD	68.9 (62.2–74.2)	12.3 (9.6–13.8)						
Lazertinib (YH25448)	Yuhan Coporation	1/2	NCT03046992		*EGFR*+ NSCLC, after previous EGFR TKI	127	Lazertinib 20, 40, 80, 120, 160, 240, 320 QD (RP2D: 240 mg QD)	54 (46–63)					Active, not recruiting	[5]	
Naquotinib (ASP8273)	Astellas Pharma	3	NCT02588261	SOLAR	*EGFR*+ NSCLC, 1L treatment	530	Arm 1: Naquotinib 300 mg QD	33 (27.4–39.0)	9.3 (5.6–11.1)	1,611	0.992		Terminated	[6]	Stopped in 2017 due to lower efficacy than osimertinib
							Arm 2: Gefitinib 250 mg daily or erlotinib 150 mg QD	47.9 (41.7–54.1)	9.6 (8.8-NE)						
Nazartinib (EGF-816)	Novartis Oncology	1/2	NCT02108964	CEGF816X2101	*EGFR*+ NSCLC, ≥ 1L treatment	180	Nazartinib 75, 100, 150, 200, 225, 300, 350 mg QD (RP2D: 150 mg QD)	51 (43–59)	9.1 (7.3–11.1)				Active, not recruiting	[7]	Stopped in 2018 due to lower efficacy and higher toxicity than osimertinib

Olmutinib (HM61713/BI 1482694)	Hanmi Pharmaceutical	1/2	NCT01588145		*EGFR* T790M+ NSCLC, after previous EGFR TKI	272	Olmutinib 300 mg QD, 500 mg BID, 800 mg QD (RP2D: 800 mg QD)	55.1 (42.6–67.1)			6.9 (5.6-9.7)			NR			Completed	[8]	Stopped in 2016 because of two cases of toxic epidermal necrolysis, one of them fatal
Osimertinib (AZD9291)	Astra Zeneca	1	NCT01802632	AURA	*EGFR* T790M+ NSCLC, after previous EGFR TKI	253	Osimertinib 20 mg QD and then escalation to 240 mg QD according to tolerance	61 (52–70) in patients with T790M+, 21 (12–34) in patients with T790M- who could be evaluated			9.6 (8.3-NR) in patients with T790M+, 2.8 (2.1–4.3) in patients with T790M-						Active, not recruiting	[9]	
		2	NCT01802632	AURA	*EGFR* T790M+ NSCLC, after previous EGFR TKI	201	Osimertinib 20, 40, 80, 160, 240 mg QD (RP2D: 80 mg QD)	62 (54–68)			12.3 (9.5–13.8)						Active, not recruiting	[10]	
		1/2	NCT01802632	AURA	*EGFR*+ NSCLC, 1L treatment	60	Osimertinib 80 or 160 mg QD	77 (64–87)			20.5 (15.0–26.1)						Active, not recruiting	[11]	
		2	NCT02094261	AURA2	*EGFR* T790M+ NSCLC, after previous EGFR TKI	210	Osimertinib 80 mg QD	70 (64–77)			9.9 (8.5–12.3)						Active, not recruiting	[12]	
		3	NCT02151981	AURA3	*EGFR* T790M+ NSCLC, after previous EGFR TKI	279	Arm 1: Osimertinib 80 mg QD	71 (65–76)	5.39 (3.47–8.48)	< 0.001	10.1	0.30 (0.23-0.41)	< 0.001	26.8 (23.5–31.5)	0.87 (0.67–1.12)	0.277	Active, not recruiting	[13,14]	
							Arm 2: Platinum-pemetrexed	31 (24–40)			4.4			22.5 (20.2–28.8)					
		3	NCT02296125	FLAURA	*EGFR*+ NSCLC, 1L treatment	556	Arm 1: Osimertinib 80 mg QD	80 (75–85)	1.27 (0.85–1.90)	0.24	18.9	0.46 (0.37–0.57)	< 0.001	38.6 (34.5-41.8)	0.80 (0.64-1.00)	0.046	Active, not recruiting	[15,16]	

							Arm 2: Gefitinib 250 mg QD or erlotinib 150 mg QD	76 (70–81)	10.2	31.8 (26.6–36)			
Rezivertinib (BPI-7711)	Beta Pharma Inc.	1	NCT03386955		*EGFR* T790M+ NSCLC, after previous EGFR TKI	82	Rezivertinib 30, 60, 120, 180, 240, 300 mg QD (RP2D: 180 mg QD)	54.5 (30–55)			Active, not recruiting	[17]	
		2	NCT03812809		*EGFR* T790M+ NSCLC, after previous EGFR TKI	226	Rezivertinib 180 mg QD				Active, not recruiting		
Rociletinib (CO-1686)	Clovis Oncology	1/2	NCT01526928	TIGER-X	*EGFR* T790M+ NSCLC, after previous EGFR TKI	130	Rociletinib 150 mg QD to 900 mg BID (RP2D: 625 mg BID)	59 (45–73) in 46 patients with T790M+, 29 (8–51) in 17 patients with T790M- who could be evaluated			Terminated	[18]	Stopped in 2016 due to lower efficacy and higher toxicity than osimertinib
TAS-121	Taiho Pharmaceutical	1	NCT02274337		*EGFR*+ NSCLC, after previous EGFR TKI	134	TAS-121 4, 8, 10, 12, 16 mg QD	28 in patients with T790M+, 19 in patients with T790M-				[19]	Stopped in 2018 due to lower efficacy than osimertinib
**BRAF +/- MEK kinase inhibitors**													
Dabrafenib (GSK-2118436) + trametinib (GSK1120212)	GlaxoSmithKline	2	NCT01336634		*BRAF* V600E+ NSCLC, after previous 1 to 3L chemotherapy	57	Dabrafenib 150 mg BID + trametinib 2 mg QD	63.2 (43.3–75.6)	9.7 (6.9–19.6)	Immature	Active, not recruiting	[20]	
		2	NCT01336634		*BRAF* V600E+	36	Dabrafenib 150 mg	64 (46–79)	10.9 (7.0–16.6)	24.6 (12.3-NE)	Completed	[21]	

				NSCLC, 1L treatment		BID+ trametinib 2 mg QD					
Vemurafenib (RO5185426, PLX4032)	Roche and Plexxicon	2	NCT01524978	*BRAF* V600+ NSCLC, ≥ 1L treatment	62	Vemurafenib 360 mg BID	37.1 (25.2–50.3) overall, 37.5 (8.5–75.5) in previously untreated patients, 37.0 (24.3–51.3) in previously treated patients	6.5 (5.29.0)	15.4 (9.6–22.8)	Completed	[22]

Table S2. Main clinical trials ongoing or planned with third- or fourth-generation EGFR tyrosine kinase inhibitors and BRAF + MEK inhibitors in advanced-stage NSCLC.

**Compound**	**Company**	**Clinical trial**						
**caption**	**caption**	**Phase**	**Clinical Trials.gov identifier**	**Name**	**Indication**	**N estimated**	**Drugs**	**Recruitement status**
				**Third-generation EGFR TKIs**				
Abivertinib (AC0010)	Hangzhou ACEA Pharmaceutical Research Co.	3	NCT03856697	AEGIS-2	*EGFR*+ NSCLC, 1L treatment	406	Arm 1: Abivertinib 300 mg BID	Not yet recruiting
							Arm 2: Gefitinib 250 mg QD	
Alflutinib (AST2818)	Shanghai Allist Pharmaceuticals	3	NCT03787992	FLAG	*EGFR*+ NSCLC, 1L treatment	358	Arm 1: Alflutinib 80 mg QD	Active, not recruiting
							Arm 2: Gefitinib 250 mg QD	
Almonertinib (HS-10296)	Jiangsu Hansoh Pharmaceutical Co.	3	NCT03849768		*EGFR*+ NSCLC, 1L treatment	350	Arm 1: Almonertinib 110 mg QD	Recruiting
							Arm 2: Gefitinib 250 mg QD	
		3	NCT04500704	ACROSS 1	*EGFR*+ NSCLC with other co-occurring driver mutations, 1L treatment	166	Arm 1: Almonertinib 110 mg QD	Not yet recruiting
							Arm 2: Almonertinib 110 mg QD+ 4 to 6 cycles of carboplatin-pemetrexed followed by pemetrexed maintenance	
		3	NCT04500717	ACROSS 2	*EGFR*+ NSCLC with co-occurring tumor suppressor genes (P53, RB1, PTEN), 1L treatment	460	Arm 1: Almonertinib 110 mg QD	Not yet recruiting
							Arm 2: Almonertinib 110 mg QD + 4 to 6 cycles of carboplatin-pemetrexed followed by pemetrexed maintenance	
ASK120067	Jiangsu Aosaikang Pharmaceutical Co.	1/2	NCT03502850		*EGFR* T790M+ NSCLC, after previous EGFR TKI	507	ASK120067 40,80,160,240,320,480 mg QD	Recruiting
		3	NCT04143607		*EGFR*+ NSCLC, 1L treatment	334	Arm 1: ASK120067 160 mg BID	Recruiting
							Arm 2: Gefitinib 250 mg QD	

D-0316	Beta Pharmaceuticals Co.	1	NCT03452150		*EGFR*+ NSCLC, after previous EGFR TKI	50	D-0316 QD, escalation according to tolerance	Active, not recruiting
		2	NCT03861156		*EGFR* T790M+ NSCLC, after previous EGFR TKI	286	D-0316 75 mg QD, escalation to 100 mg QD according to tolerance	Active, not recruiting
		2/3	NCT04206072		*EGFR*+ NSCLC, 1L treatment	360	Arm 1: D-0316 75 mg QD for first cycle and then escalation to 100 mg QD according to tolerance<br>Arm 2: Icotinib 125 mg TID	Active, not recruiting
Lazertinib (YH25448)	Yuhan Coporation	3	NCT04248829	LASER301	*EGFR*+ NSCLC, 1L treatment	380	Arm 1: Lazertinib 240 mg QD<br>Arm 2: Gefitinib 250 mg QD	Recruiting
		3	NCT04487080	MARIPOSA	*EGFR*+ NSCLC, 1L treatment	1000	Arm 1: Lazertinib 240 mg QD + amivantamab (IV) (1050 mg if < 80 kg and 1400 mg if ≥ 80 kg in 28-day cycles: once weekly in cycle 1 (split dose on days 1-2) and then every 2 weeks)<br>Arm 2: Lazertinib 240 mg QD + matching osimertinib placebo<br>Arm 3: Osimertinib 80 mg QD + matching lazertinib placebo	Recruiting
Osimertinib (AZD9291)	Astra Zeneca	3	NCT04035486	FLAURA2	*EGFR*+ NSCLC, 1L treatment	586	Arm 1: Osimertinib 80 mg QD<br>Arm 2: Osimertinib 80 mg QD + 4 cycles of platinum-pemetrexed followed by pemetrexed maintenance	Recruiting
Rezivertinib (BPI-7711)	Beta Pharma Inc.	3	NCT03866499	RAZOR	*EGFR*+ NSCLC, 1L treatment	294	Arm 1: Rezivertinib 180 mg QD<br>Arm 2: Gefitinib 250 mg QD	Recruiting
SH-1028	Nanjing Sanhome Pharmaceutical Co.	1	NCT03603262		*EGFR*+ NSCLC, after previous EGFR TKI	85	SH-1028 starting dose 60 mg QD. If tolerated subsequent cohorts will test increasing doses: 100,200,300,400 mg QD	Unknown
		2	NCT03823807		*EGFR* T790M+ NSCLC, after previous EGFR TKI	300	SH-1028 100 mg QD	Unknown
		3	NCT04239833		*EGFR*+ NSCLC, 1L treatment	245	Arm 1: SH-1028 200 mg QD<br>Arm 2: Gefitinib 250 mg QD	Not yet recruiting

				**Fourth-generation EGFR TKIs**				
BLU-945	Blueprint Medicines	1/2	NCT04862780	SYMPHONY	*EGFR* T790M and C797S+ NSCLC, after previous EGFR TKI	120	BLU-945, escalation according to tolerance	Recruiting
				**BRAF + MEK kinase inhibitors**				
Dabrafenib (GSK-2118436) + trametinib (GSK1120212)	GlaxoSmithKline	2	NCT03543306	BRF113928	*BRAF* V600E+ NSCLC, after previous 1 to 2L chemotherapy	27	Dabrafenib 150 mg BID + trametinib 2 mg QD	Recruiting
Encorafenib (LGX818) + binimetinib (ARRY-162)	Novartis, Array Biopharma	2	NCT03915951		*BRAF* V600E+ NSCLC, ≥ 1L treatment	90	Encorafenib 450 mg QD + binimetinib 45 mg BID	Recruiting

References

1. Ma, Y.; Zheng, X.; Zhao, H.; Fang, W.; Zhang, Y.; Ge, J.; Wang, L.; Wang, W.; Jiang, J.; Chuai, J. et al. First-in-Human Phase I Study of AC0010, a Mutant-Selective EGFR Inhibitor in Non-Small Cell Lung Cancer: Safety, Efficacy, and Potential Mechanism of Resistance. *J. Thorac. Oncol.* **2018**, *13*, 968–977.
2. Shi, Y.; Zhang, S.; Hu, X.; Feng, J.; Ma, Z.; Zhou, J.; Yang, N.; Wu, L.; Liao, W.; Zhong, D.; et al. Safety, Clinical Activity, and Pharmacokinetics of Alflutinib (AST2818) in Patients With Advanced NSCLC With EGFR T790M Mutation. J. Thorac. Oncol. **2020**, *15* 1015–1026.
3. Shi, Y.; Hu, X.; Zhang, S.; Lv, D.; Wu, L.; Yu, Q.; Zhang, Y.; Liu, L.; Wang, X.; Cheng, Y.; et al. Efficacy, safety, and genetic analysis of furmonertinib (AST2818) in patients with EGFR T790M mutated non-small-cell lung cancer: a phase 2b, multicentre, single-arm, open-label study. *Lancet. Respir. Med*. **2021**, *9*, 829–839.
4. Yang, J.C.; Camidge, D.R.; Yang, C.T.; Zhou, J.; Guo, R.; Chiu, C.H.; Chang, G.C.; Shiah, H.S.; Chen, Y.; Wang, C.C.; et al. Safety, Efficacy, and Pharmacokinetics of Almonertinib (HS-10296) in Pretreated Patients With EGFR-Mutated Advanced NSCLC: A Multicenter, Open-label, Phase 1 Trial. *J. Thorac. Oncol.* **2020**, *15*, 1907–1918.
5. Ahn, M.J.; Han, J.Y.; Lee, K.H.; Kim, S.W.; Kim, D.W.; Lee, Y.G.; Cho, E.K.; Kim, J.H.; Lee, G.W.; Lee, J.S.; et al. Lazertinib in patients with EGFR mutation-positive advanced non-small-cell lung cancer: results from the dose escalation and dose expansion parts of a first-in-human, open-label, multicentre, phase 1-2 study. *Lancet. Oncol*. **2019**, *20*, 1681–1690.
6. Kelly, R.J.; Shepherd, F.A.; Krivoshik, A.; Jie, F.; Horn, L. A phase III, randomized, open-label study of ASP8273 versus erlotinib or gefitinib in patients with advanced stage IIIB/IV non-small-cell lung cancer. *Ann. Oncol.* **2019**, *30*, 1127–1133.
7. Tan, D.S.; Leighl, N.B.; Riely, G.J.; Yang, J.C.; Sequist, L.V.; Wolf, J.; Seto, T.; Felip, E.; Aix, S.P.; Jonnaert, M.; et.al. Safety and efficacy of nazartinib (EGF816) in adults with EGFR-mutant non-small-cell lung carcinoma: a multicentre, open-label, phase 1 study. *Lancet. Respir. Med.* **2020**, *8*, 561–572.
8. Kim, D.W.; Lee, D.H.; Han, J.Y.; Lee, J.; Cho, B.C.; Kang, J.H.; Lee, K.H.; Cho, E.K.; Kim, J.S.; Min, Y.J.; et al. Safety, tolerability, and anti-tumor activity of olmutinib in non-small cell lung cancer with T790M mutation: A single arm, open label, phase 1/2 trial. *Lung Cancer* **2019**, *135*, 66–72.
9. Jänne, P.A.; Yang, J.C.; Kim, D.W.; Planchard, D.; Ohe, Y.; Ramalingam, S.S.; Ahn, M.J.; Kim, S.W.; Su, W.C.; Horn, L.; et al. AZD9291 in EGFR inhibitor-resistant non-small-cell lung cancer. *N. Engl. J. Med.* **2015**, *372*, 1689–99.
10. Yang, J.C.; Ahn, M.J.; Kim, D.W.; Ramalingam, S.S.; Sequist, L.V.; Su, W.C.; Kim, S.W.; Kim, J.H.; Planchard, D.; Felip, E.; et al. Osimertinib in Pretreated T790M-Positive Advanced Non-Small-Cell Lung Cancer: AURA Study Phase II Extension Component. *J. Clin. Oncol.* **2017**, *35*, 1288–1296.
11. Ramalingam, S.S.; Yang, J.C.; Lee, C.K.; Kurata, T.; Kim, D.W.; John, T.; Nogami, N.; Ohe, Y.; Mann, H.; Rukazenkov, Y.; et al. Osimertinib As First-Line Treatment of EGFR Mutation-Positive Advanced Non-Small-Cell Lung Cancer. *J. Clin. Oncol.* **2018**, *36*, 841–849.
12. Goss, G.; Tsai, C.M.; Shepherd, F.A.; Bazhenova, L.; Lee, J.S.; Chang, G.C.; Crino, L.; Satouchi, M.; Chu, Q.; Hida, T.; et al. Osimertinib for pretreated EGFR Thr790Met-positive advanced non-small-cell lung cancer (AURA2): a multicentre, open-label, single-arm, phase 2 study. *Lancet. Oncol.* **2016**, *17*, 1643–1652.

13. Mok, T.S.; Wu, Y.L.; Ahn, M.J.; Garassino, M.C.; Kim, H.R.; Ramalingam, S.S.; Shepherd, F.A.; He, Y.; Akamatsu, H.; Theelen, W.S.; et al. Osimertinib or Platinum-Pemetrexed in EGFR T790M-Positive Lung Cancer. *N. Engl. J. Med.* **2017**, *376*, 629–640.
14. Papadimitrakopoulou, V.A.; Mok, T.S.; Han, J.Y.; Ahn, M.J.; Delmonte, A.; Ramalingam, S.S.; Kim, S.W.; Shepherd, F.A.; Laskin, J.; He, Y.; Akamatsu, H.; et al. Osimertinib versus platinum-pemetrexed for patients with EGFR T790M advanced NSCLC and progression on a prior EGFR-tyrosine kinase inhibitor: AURA3 overall survival analysis. *Ann. Oncol.* **2020**, *31*, 1536–1544.
15. Ramalingam, S.S.; Vansteenkiste, J.; Planchard, D.; Cho, B.C.; Gray, J.E.; Ohe, Y.; Zhou, C.; Reungwetwattana, T.; Cheng, Y.; Chewaskulyong, B.; et.al. Overall Survival with Osimertinib in Untreated, EGFR-Mutated Advanced NSCLC. *N. Engl. J. Med.* **2020**, *382*, 41–50.
16. Soria, J.C.; Ohe, Y.; Vansteenkiste, J.; Reungwetwattana, T.; Chewaskulyong, B.; Lee, K.H.; Dechaphunkul, A.; Imamura, F.; Nogami, N.; Kurata, T.; Okamoto, I.; et al. Osimertinib in Untreated EGFR-Mutated Advanced Non-Small-Cell Lung Cancer. *N. Engl. J. Med*. **2018**, *378*, 113–125.
17. Shi, Y.; Fang, J.; Shu, Y.; Wang, D.; Yu, H.; Zhao, Y.; Zhang, L.; Zhu, B.; Li, X.; Chen, G.; Shi, J.; Zheng, R.; et al, A phase 1 study to evaluate safety and antitumor activity of BPI-7711 in EGFRM+/T790M+ advanced or recurrent NSCLC patients. *J. Thorac. Oncol.* **2019**, *37*, 9034.
18. Sequist, L.V.; Soria, J.C.; Goldman, J.W.; Wakelee, H.A.; Gadgeel, S.M.; Varga, A.; Papadimitrakopoulou, V.; Solomon, B.J.; Oxnard, G.R.; Dziadziuszko, R.; et al. Rociletinib in EGFR-mutated non-small-cell lung cancer. *N. Engl. J. Med.* **2015**, *372*, 1700–9.
19. Nishio, M.; Murakami, H.; Ohe, H.; Hida, T.; Sakai, H.; Kasahara, K.; Imamura, F.; Baba, T.; Kubota, K.; Hosomi, Y.; et. al. Phase I study of TAS-121, a third-generation epidermal growth factor receptor (EGFR) tyrosine kinase inhibitor, in patients with non-small-cell lung cancer harboring EGFR mutations. *Invest. New Drugs* **2019**, *37*, 1207–1217.
20. Planchard, D.; Besse, B.; Groen, H.J.M.; Souquet, P.J.; Quoix, E.; Baik, C.S.; Barlesi, F.; Kim, T.M.; Mazieres, J.; Novello, S.; et al. Dabrafenib plus trametinib in patients with previously treated BRAF(V600E)-mutant metastatic non-small cell lung cancer: an open-label, multicentre phase 2 trial. *Lancet. Oncol*. **2016**, *17*,984–993.
21. Planchard, D.; Smit, E.F.; Groen, H.J.M.; Mazieres, J.; Besse, B.; Helland, Å.; Giannone, V.; D'Amelio, A.M.; Zhang, Jr.P.; Mookerjee, B.; et al. Dabrafenib plus trametinib in patients with previously untreated BRAF(V600E)-mutant metastatic non-small-cell lung cancer: an open-label, phase 2 trial. *Lancet. Oncol*. **2017**, *18*, 1307–1316.
22. Subbiah, V.; Gervais, R.; Riely, G.; Hollebecque, A.; Blay, J.Y.; Felip, E.; Schuler, M.; Gonçalves, A.; Italiano, A.; Keedy, V.; et al. Efficacy of Vemurafenib in Patients With Non-Small-Cell Lung Cancer With BRAF V600 Mutation: An Open-Label, Single-Arm Cohort of the Histology-Independent VE-BASKET Study. *JCO Precis. Oncol.* **2019**, *3*
